# Antagonism between Front-Line Antibiotics Clarithromycin and Amikacin in the Treatment of Mycobacterium abscessus Infections Is Mediated by the *whiB7* Gene

**DOI:** 10.1128/AAC.01353-17

**Published:** 2017-10-24

**Authors:** Mark Pryjma, Ján Burian, Kevin Kuchinski, Charles J. Thompson

**Affiliations:** Department of Microbiology and Immunology and the Centre for Tuberculosis Research, University of British Columbia, Vancouver, Canada

**Keywords:** Mycobacterium abscessus, drug resistance, antagonize, synergize, *whiB7*, macrolide, aminoglycoside, clarithromycin, amikacin, cystic fibrosis, antibiotic resistance, synergy

## Abstract

Combinations of antibiotics, each individually effective against Mycobacterium abscessus, are routinely coadministered based on the concept that this minimizes the spread of antibiotic resistance. However, our *in vitro* data contradict this assumption and instead document antagonistic interactions between two antibiotics (clarithromycin and amikacin) used to treat M. abscessus infections. Clinically relevant concentrations of clarithromycin induced increased resistance to both amikacin and itself. The induction of resistance was dependent on *whiB7*, a transcriptional activator of intrinsic antibiotic resistance that is induced by exposure to many different antibiotics. In M. abscessus, the deletion of *whiB7* (MAB_3508c) resulted in increased sensitivity to a broad range of antibiotics. WhiB7 was required for transcriptional activation of genes that confer resistance to three commonly used anti-M. abscessus drugs: clarithromycin, amikacin, and tigecycline. The *whiB7*-dependent gene that conferred macrolide resistance was identified as *erm*(41) (MAB_2297), which encodes a ribosomal methyltransferase. The *whiB7*-dependent gene contributing to amikacin resistance was *eis2* (MAB_4532c), which encodes a Gcn5-related *N*-acetyltransferase (GNAT). Transcription of *whiB7* and the resistance genes in its regulon was inducible by subinhibitory concentrations of clarithromycin but not by amikacin. Thus, exposure to clarithromycin, or likely any *whiB7*-inducing antibiotic, may antagonize the activities of amikacin and other drugs. This has important implications for the management of M. abscessus infections, both in cystic fibrosis (CF) and non-CF patients.

## INTRODUCTION

Mycobacterium abscessus is a rapidly growing, intrinsically drug-resistant, nontuberculous mycobacterium that has become a global health problem. A recent epidemiological study documented its prevalence and transmission between hospital settings throughout the world, suggesting that M. abscessus has become a resident hospital pathogen rather than an opportunistic pathogen (1). Numerous local outbreaks of M. abscessus infections were described in 2016 (1–5). In addition to invading patients with inflammatory lung diseases, such as cystic fibrosis (6), M. abscessus causes serious cutaneous, joint, soft tissue, surgical site, and disseminated infections (7).

The fact that M. abscessus is among the most difficult to treat mycobacterial infections has accelerated its spread and evolution as a dangerous pathogen (8, 9). It is resistant to most antibiotics (10), including those commonly used to treat Mycobacterium tuberculosis infections (rifampin, isoniazid, and ethambutol). The only clinically available antibiotics known to have significant *in vitro* activity against M. abscessus are clarithromycin (a macrolide), amikacin (an aminoglycoside), cefoxitin (a cephalosporin), and imipenem (a carbapenem) (7). Clarithromycin is the cornerstone for M. abscessus therapy and is routinely coadministered in combinations with amikacin, cefoxitin, imipenem, or tigecycline for 6 to 12 months. However, treatment outcome is unpredictable, and many cases are untreatable (11–14).

This serious problem has motivated studies that have revealed a complex overlapping network of resistance mechanisms (15). In both M. abscessus and M. tuberculosis, high levels of intrinsic antibiotic resistance are provided, in part, by their low-permeability cell envelopes (10, 16, 17). In addition, M. tuberculosis upregulates the expression of resistance genes (15, 18–20) able to combat internalized antibiotics. These genes include *eis* (encoding an acetyltransferase that modifies aminoglycosides [18, 19, 21]), *erm* (encoding a ribosomal methyltransferase that prevents macrolide binding [22]), and *tap* (encoding an efflux pump able to export drugs, including aminoglycosides, tetracyclines, and *para*-aminosalicylic acid [23]). Although these genes all encode proteins that can provide resistance to certain antibiotics, increased expression is needed to optimize their activities. Importantly, all of these resistance genes are upregulated by WhiB7, a transcriptional activator that is conserved across mycobacteria and related actinomycetes (18–20, 24).

The WhiB7-dependent network of resistance genes is activated by environmental stress signals and a broad range of antibiotics, including macrolides, lincosamides, aminoglycosides, tetracyclines, and pleuromutilins (18–20, 25). Importantly, the induction of WhiB7-mediated resistance by one type of inducer can promote cross-resistance to other types of antibiotics (18). Recently published M. abscessus data revealed that an orthologous WhiB7 regulon (including *erm* and *tap* identified in Mycobacterium smegmatis and M. tuberculosis) was upregulated in response to the macrolide erythromycin (26), a known inducer of WhiB7-mediated resistance in other Mycobacterium species (18). In fact, an *erm* ortholog [*erm*(41)] has been identified as an inducible macrolide resistance gene in M. abscessus (27, 28).

Since M. abscessus therapy typically includes the macrolide clarithromycin, a *whiB7* inducer, we speculated that it might affect the M. abscessus antibiotic resistance spectra (specifically, aminoglycoside resistance). Exposure to clarithromycin should increase the expression of the macrolide resistance gene *erm*(41) and of potential aminoglycoside resistance genes (including the M. abscessus ortholog of *tap*). Studies reported here show that exposure to clarithromycin induces intrinsic aminoglycoside resistance in a WhiB7-dependent manner. The activation of cross-resistance to amikacin by clarithromycin has important clinical implications for treating M. abscessus infections, including those in patients with cystic fibrosis.

## RESULTS

### Clarithromycin induces resistance to amikacin.

Clarithromycin is known to induce expression of the M. abscessus *erm*(41) gene, which results in increased macrolide resistance (28). To determine whether clarithromycin induced resistance to other antibiotics, M. abscessus ATCC 19977 cultures were incubated in ½ or ¼ the MIC of clarithromycin for 24 h, followed by the addition of antibiotics used to treat M. abscessus infections (amikacin, imipenem, cefoxitin, or tigecycline). Growth inhibition was determined using the resazurin colorimetric assay to establish the MIC. Preincubation with clarithromycin resulted in a 4-fold increase in amikacin MIC (from 3.1 to 12.5 mg/liter) (Table 1) but caused no change in the MICs of imipenem, cefoxitin, or tigecycline (data not shown). Corresponding studies were done using six independently isolated clinical strains of M. abscessus (Table 1). Five of the six strains (strains no. 2 to 6) displayed a 4-fold increase in amikacin MIC when preincubated with clarithromycin; strain no. 1 had high levels of constitutive amikacin resistance without preincubation, likely due to mutation(s) in its 16S rRNA (29). Increased amikacin resistance resulting from clarithromycin exposure occurred rapidly; a 4-fold increase in amikacin MIC took place with 1 h of preincubation (data not shown).

**TABLE 1:** Amikacin resistance of M. abscessus strains preincubated with subinhibitory concentrations of clarithromycin

Clarithromycin concn (mg/liter)	Amikacin MIC (mg/liter) by strain^*a*^						
	ATCC 19977	No. 1	No. 2	No. 3	No. 4	No. 5	No. 6
0	3.1	>50	3.1	3.1	3.1	3.1	3.1
0.05	12.5	>50	12.5	12.5	12.5	12.5	12.5
0.1	12.5	>50	12.5	12.5	12.5	12.5	12.5

a Values are the median values of 3 experiments.

**FIG 1:**
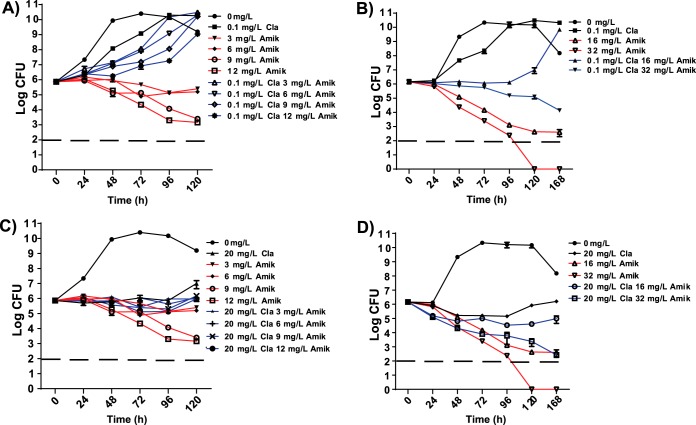
Effect of clarithromycin preincubation on amikacin bactericidal activity. WT M. abscessus was untreated or preincubated with clarithromycin (Cla) at sub-MIC (0.1 mg/liter) (A and B) or 100× the MIC (20 mg/liter) (C and D) for 2.5 h. Following preincubation, amikacin (Amik) was added to the mixture described in panels A and C at 3 to 12 mg/liter, or in panels B and D at 16 to 32 mg/liter. CFU were determined at 24-h intervals post-amikacin addition. The dashed line represents the limit of detection. Data points are the mean of 3 duplicates, with standard deviation presented as error bars.

Unlike amikacin, which is bactericidal, clarithromycin is bacteriostatic at the clinically relevant concentrations used in our studies. To test the effect of clarithromycin preincubation on the bactericidal effect of amikacin, cultures were exposed for 2.5 h to clarithromycin concentrations that were subinhibitory for growth (0.1 mg/liter; ½ the MIC) (Fig. 1A and B) or to therapeutic concentrations found in the lung (20 mg/liter; 100× the MIC) (Fig. 1C and D). The cultures were then challenged using a range of amikacin concentrations, including those found in the lungs during amikacin therapy (3 to 12 mg/liter; Fig. 1A and C), as well as those used to define sensitivity (<16 mg/liter) or intermediate resistance (32 mg/liter [30]) (Fig. 1B and D). Amikacin bactericidal activity was recorded as the change in the number of CFU over 4 to 6 days. Without clarithromycin preincubation, low concentrations of amikacin (3 to 6 mg/liter) prevented increases in the number of viable cells, and higher concentrations (9 to 32 mg/liter) reduced the number of CFU in a time- and concentration-dependent manner (Fig. 1A and B). Even at the lowest amikacin concentration tested (3 mg/liter), no increase in CFU occurred over the time period (0 to 120 h). However, after preincubation with ½ the MIC of clarithromycin, bacterial growth occurred at amikacin concentrations up to 16 mg/liter (Fig. 1A and B), and the bactericidal activity of 32 mg/liter amikacin was noticeably reduced (Fig. 1B). After preincubation in 100× the MIC clarithromycin (Fig. 1C and D), the bactericidal activity of amikacin in the 3 to 12 mg/liter range was completely suppressed (Fig. 1C), and its bactericidal activity at higher concentrations (16 to 32 mg/liter) was markedly reduced (Fig. 1D). Without preincubation, inhibition of amikacin bactericidal activity by clarithromycin occurred to a lesser extent (see Fig. S2 in the supplemental material).

### *whiB7* was essential for resistance to a broad spectrum of antibiotics.

**FIG 2:**
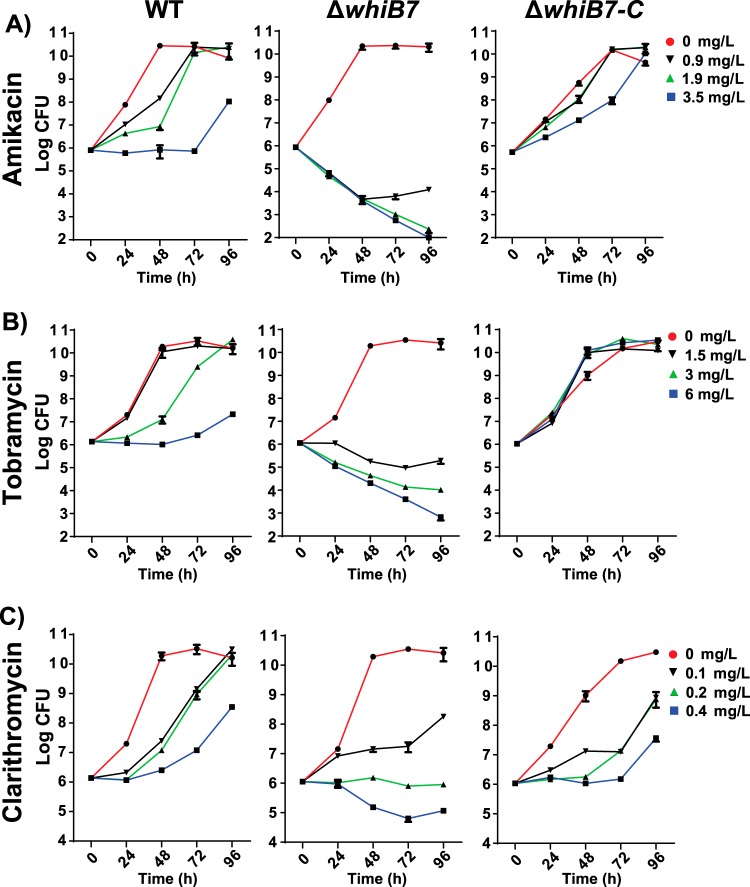
Effect of deleting *whiB7* on amikacin, tobramycin, and clarithromycin resistance. WT (left), Δ*whiB7* mutant (middle), and Δ*whiB7*-C (right) cultures were incubated in amikacin (A), tobramycin (B), or clarithromycin (C), at the indicated concentrations. CFU were monitored at 24-h intervals. Data points are the means of 3 replicates, with standard deviations shown as error bars. The data represent 3 independent experiments.

In other mycobacteria, WhiB7 is a central regulator of multidrug resistance, including inducible macrolide resistance (18, 24). We constructed a deletion of the *whiB7* homolog in M. abscessus (MAB_3508c) and analyzed its resistance using both MIC and kill curve (CFU) assays. The MIC data showed that resistance to a variety of antibiotics was decreased in the Δ*whiB7* mutant compared to the wild type (Table 2). The Δ*whiB7* mutant was more sensitive to aminoglycosides (tobramycin, gentamicin, sisomicin, and amikacin), phenicols (chloramphenicol), tetracyclines (tigecycline), and clarithromycin than the wild type. Sensitivity to imipenem, cefoxitin, doxycycline, tetracycline, clindamycin, levofloxacin, moxifloxacin, rifabutin, rifamycin, isoniazid, or ethambutol was unaltered (Table S2). Kill curve analyses of tobramycin, amikacin, and clarithromycin against wild-type (WT) and Δ*whiB7* mutant strains demonstrated increased activities of all antibiotics against the mutant Δ*whiB7* (Fig. 2). Resistance was restored when the *whiB7* mutant was complemented (Δ*whiB7*-C; Fig. 2). For both aminoglycosides (amikacin and tobramycin), all concentrations tested allowed growth of the wild type (at reduced rates) but were bactericidal for the Δ*whiB7* mutant (Fig. 2A and B). Importantly, clarithromycin prevented Δ*whiB7* mutant strain CFU increases at 0.2 or 0.4 mg/liter, whereas WT CFU continuously increased at these concentrations (Fig. 2C). Clinically relevant concentrations of clarithromycin (3 to 20 mg/liter) caused much higher rates of killing in the Δ*whiB7* mutant (Fig. S3).

**TABLE 2:** Effect of *whiB7* deletion on the resistance profile of M. abscessus

Antibiotic	MIC (mg/liter)^*a*^			
	0 mg/liter clarithromycin		½ the MIC of clarithromycin	
	WT	Δ*whiB7* mutant	WT	Δ*whiB7* mutant
Amikacin	3.1	0.8	12.5	0.8
Tobramycin	6.3	1.6	25	1.6
Gentamicin	6.3	1.6	25	1.6
Sisomicin	3.1	0.8	12.5	0.8
Chloramphenicol	100	25	100	25
Tigecycline	0.8	0.2	0.8	0.2
Clarithromycin	0.2	0.05	0.2	0.05
Clarithromycin^*b*^	3.1	0.1	25	0.4

a Values are the medians of 3 experiments.

b Clarithromycin resistance was also measured after 7 days of incubation, which is standard for macrolide MIC analysis.

### Role of *whiB7* in clarithromycin-induced activation of amikacin resistance.

Studies were done to determine whether clarithromycin-induced activation of amikacin resistance was dependent on WhiB7. Cultures were untreated or preincubated with clarithromycin at ½ the MIC (relative to their respective MICs) for 24 h and assayed for amikacin resistance. Without clarithromycin preinduction, the Δ*whiB7* mutant had a 4-fold reduction in amikacin MIC compared to the WT (3.1 to 0.8 mg/liter; Table 2). Preincubation with clarithromycin resulted in a 4-fold increase in WT amikacin MIC but had no effect on Δ*whiB7* mutant amikacin resistance. Additionally, induction of amikacin resistance was not observed in cultures of the Δ*whiB7* mutant preincubated with a concentration of clarithromycin that induced resistance in the WT (0.1 mg/liter; data not shown). Preincubation with clarithromycin also generated a *whiB7*-dependent 4-fold increase in MIC of other aminoglycosides (tobramycin, gentamicin, and sisomicin; Table 2).

To examine whether additional antibiotics induced amikacin resistance in a *whiB7*-dependent manner, WT and Δ*whiB7* mutant cultures were preincubated for 24 h with ½ the MIC of clarithromycin, chloramphenicol, tigecycline, or amikacin. Clarithromycin and tigecycline induced a 4-fold increase in amikacin resistance, while chloramphenicol induced a 2-fold increase in the WT but not in the Δ*whiB7* mutant (Table 3); amikacin, however, did not induce resistance to itself in either strain (Table 3). Preincubation with cefoxitin or imipenem, two other drugs commonly used to control M. abscessus, did not induce amikacin resistance (data not shown).

**TABLE 3:** Amikacin resistance is affected by preincubation with subinhibitory concentrations of various antibiotics in M. abscessus WT and Δ*whiB7* mutant

Preincubation with ½ the MIC	Amikacin MIC (mg/liter)^*a*^	
	WT	Δ*whiB7* mutant
No drug	3.1	0.8
Clarithromycin	12.5	0.8
Amikacin	3.1	0.8
Chloramphenicol	6.1	0.4
Tigecycline	12.5	0.1

a Values are the medians of 3 experiments.

### Clarithromycin induced a *whiB7*-dependent increase in clarithromycin resistance.

Previous studies showed that *erm*(41) is responsible for clarithromycin-induced macrolide resistance (28); *erm*(41) transcription increases for 24 h, followed by increased resistance to clarithromycin within the first week. Therefore, we exposed cultures to clarithromycin for 24 h and then measured the clarithromycin MIC after 7 days to assess the consequences of *whiB7* disruption on the induction of macrolide resistance. Without preincubation, the Δ*whiB7* mutant was 8-fold more sensitive than the WT to clarithromycin (Table 2). Preinduction of the WT culture with ½ the MIC of clarithromycin induced a further 8-fold increase in resistance to clarithromycin in WT but had no effect on the Δ*whiB7* mutant MIC (Table 2). In total, the preincubated WT culture assayed at 7 days had a 64-fold increase in clarithromycin MIC relative to the Δ*whiB7* mutant. In conclusion, *whiB7*-dependent induction of clarithromycin resistance extends over many days and was a major determinant of clarithromycin resistance in M. abscessus.

### WhiB7-dependent induction of macrolide and aminoglycoside resistance genes.

**FIG 3:**
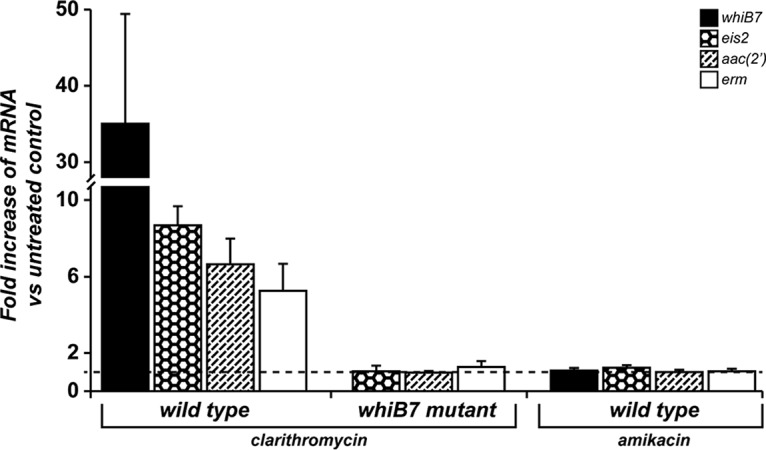
Antibiotic-induced transcription of *whiB7*, *eis2*, *aac*(*2′*), and *erm* in M. abscessus WT and a Δ*whiB7* mutant. Increases in *whiB7*, *eis2*, *aac*(*2′*), and *erm* mRNAs after 2.5 h of treatment with clarithromycin (0.1 mg/liter WT and 0.025 mg/liter Δ*whiB7* mutant) or amikacin (1.6 mg/liter) relative to untreated controls of the same strain are shown. Data are the mean of three biologically independent samples, with standard deviation as the error bars. The dashed line indicates a value (treated versus untreated ratio of 1) corresponding to no antibiotic-induced change.

We observed *whiB7*-dependent upregulation of macrolide and aminoglycoside (including amikacin) resistance in cultures preincubated with clarithromycin (Table 2). Quantitative reverse transcription-PCR (RT-qPCR) verified that WhiB7 was induced by clarithromycin and was needed for *erm*(41) induction in M. abscessus (Fig. 3). It has been established that *erm*(41) is responsible for inducible macrolide resistance in M. abscessus (28); studies in other mycobacteria (18) showed that the induction of orthologous *erm* genes is dependent on WhiB7 and causes inducible macrolide resistance. In summary, our studies showed that *erm*(41) and *whiB7* also provided inducible macrolide resistance in M. abscessus.

To identify the amikacin resistance factors, we initially focused on two possible aminoglycoside resistance genes, MAB_1409c and MAB_4395, induced by the macrolide erythromycin in M. abscessus (26). MAB_1409c is the ortholog of M. tuberculosis tap, encoding an efflux pump known to provide aminoglycoside resistance that is under WhiB7 regulation (23). MAB_4395 [*aac*(*2′*)] is annotated in online databases as an aminoglycoside 2′-*N*-acetyltransferase gene (31). These genes were disrupted and the mutants analyzed for resistance to amikacin and other representative aminoglycosides (tobramycin, sisomicin, and gentamicin). The Δ*tap* mutant displayed no significant change in resistance to any of these antibiotics (Table S3). The Δ*aac*(*2′*) mutant was more sensitive to several aminoglycosides, including tobramycin (16-fold), sisomicin (4-fold), and gentamicin (2-fold), but there were no changes in its resistance to amikacin (Table S3). Preincubation with clarithromycin increased the MICs to tobramycin, sisomicin, and gentamicin (but not amikacin) in the WT but not the Δ*aac*(*2′*) mutant (Table S3), implying that upregulation of *aac*(*2′*) accounts for resistance to some aminoglycosides but not amikacin. RT-qPCR demonstrated that Δ*aac*(*2′*) expression was indeed under WhiB7 control (Fig. 3). Additionally, amikacin resistance was induced 4-fold by clarithromycin in the Δ*aac*(*2′*) mutant, indicating that *aac*(*2′*) was not necessary for M. abscessus amikacin resistance. These results indicated that M. abscessus contained additional WhiB7-regulated genes that provide amikacin resistance.

A recently published study confirmed our observations regarding the Δ*aac*(*2′*) mutant and identified MAB_4532c (*eis2*) as an amikacin resistance determinant in M. abscessus (21, 32). We used RT-qPCR to determine whether *eis2* was in the WhiB7 regulon and could be responsible for clarithromycin-induced amikacin resistance. Indeed, *eis2* transcription was induced by clarithromycin in a WhiB7-dependent manner (Fig. 3), establishing its inclusion in the WhiB7 regulon. Consistent with the findings of resistance studies described above, this gene was not induced by amikacin (Fig. 3). These *in vitro* data provide evidence that *eis2* is likely to play an important role in WhiB7-dependent, clarithromycin-induced amikacin resistance in M. abscessus.

## DISCUSSION

Although combinations of antibiotics are routinely used to treat bacterial diseases and often have synergistic activities, there can also be antagonistic interactions (33–35). In 1952, Jawetz and Gunnison first reported that bacteriostatic antibiotics can inhibit the activities of bactericidal antibiotics (36) (see review in reference 33). We discovered a mechanism for antagonism between antibiotics currently used for M. abscessus therapy. Our studies revealed that clarithromycin, a bacteriostatic macrolide that is the cornerstone antibiotic for the treatment of M. abscessus infections, antagonized the activity of a partnered bactericidal aminoglycoside (amikacin). Based on previous studies in M. smegmatis and M. tuberculosis, we explored whether the antagonism relied on antibiotic-induced stress signals that triggered genes within the WhiB7 resistance regulon.

Treatment of M. abscessus lung and cystic fibrosis infections involves 2 to 3 antibiotics taken for up to a year. This traditional practice is costly and has numerous undesirable side effects. A recent meta-analysis and systematic review of seroconversion in pulmonary M. abscessus infections found that antibiotic treatment outcomes were successful only 41% of the time with surgical intervention and 35% of the time without surgery, concluding that most patients will retain chronic infection (37). The ineffectiveness of the standard triple therapy (clarithromycin, amikacin, and cefoxitin) in eliminating infection has been confirmed in a hollow fiber M. abscessus lung disease model (38). Following up reports that clarithromycin induced clarithromycin resistance, we explored the concept that it might also alter resistance to amikacin and other coadministered antibiotics. Our data revealed that preincubation with clarithromycin increased the amikacin MIC 4-fold and reduced or eliminated its bactericidal activity throughout a clinically relevant concentration range (3 to 32 mg/liter; Fig. 1). A review of drug concentrations in patients undergoing treatment for pneumonia showed that the amikacin maximum concentration of drug in serum (*C*_max_) in lungs reached 10 mg/liter after standard intravenous (i.v.) treatments (15 mg/kg of body weight/day dosing) (39). Even at higher i.v. doses used to treat cystic fibrosis patients (35 mg/kg/day amikacin), a *C*_max_ of only 11 mg/liter is achieved (40). This implies that clarithromycin treatment of M. abscessus may allow bacterial growth at concentrations of amikacin higher than those achieved in patients if administered as little as 1 h before amikacin. The antagonistic activities of clarithromycin and amikacin were still present, although at a lower rate, if they were added at the same time (Fig. S2). A range of other antibiotics, including chloramphenicol and tigecycline, also induced amikacin resistance (Table 3). In addition, we found that clarithromycin also increased resistance to three other aminoglycosides (Table 2). These results are clinically important proof that exposure to specific antibiotics can induce a multidrug resistance state in M. abscessus. In other mycobacteria, WhiB7 provides multidrug resistance and is upregulated in response to many different antibiotics, including a range of macrolides. We therefore genetically inactivated *whiB7* in M. abscessus to analyze its role in the response to antibiotic exposure and identified several genes in its regulon that may provide drug resistance.

WhiB7 function was required for resistance to aminoglycosides (amikacin, tobramycin, gentamicin, and sisomicin), macrolides (clarithromycin), tigecycline, and chloramphenicol (Table 2), and for resisting the bactericidal effects of clarithromycin at high concentrations (Fig. S3). It was also required for increased levels of resistance induced by preincubation with clarithromycin, tigecycline, or chloramphenicol (Table 3). Curiously, while WhiB7 was needed for resistance to amikacin, preincubation with amikacin did not amplify resistance to itself (Table 3). RT-qPCR analyses demonstrated that amikacin did not induce transcription of *whiB7* or other genes under WhiB7 control (Fig. 3). Therefore, while WhiB7 was a key player in amikacin resistance, the inability of amikacin to induce *whiB7* expression minimized amikacin's ability to increase aminoglycoside resistance. Knowing that clarithromycin induced amikacin resistance and is an established *whiB7* inducer (18), we used it to explore the WhiB7 resistance regulon in M. abscessus.

Resistance spectra, clarithromycin-induced expression, and *whiB7* dependence were analyzed for the aminoglycoside resistance genes *tap* (MAB_1409c) and *aac*(*2′*) (MAB_4395), as well as the macrolide resistance gene *erm*(41) (MAB_2297). RT-qPCR studies showed that transcription of *whiB7*, *tap*, *aac*(*2′*), and *erm*(41) was induced by clarithromycin treatment in a *whiB7*-dependent manner (Fig. 3). However, *tap* and *aac*(*2′*) (a resistance determinant for several aminoglycosides) could not be linked to amikacin resistance (Table S3).

To identify the *whiB7*-dependent mediator of amikacin resistance, we analyzed the recently reported mycobacterial amikacin resistance determinant *eis2* (MAB_ 4532c), an *eis* paralog (32). The deletion of *eis2* causes an 8-fold reduction in amikacin MIC in M. abscessus (32), and *eis2* was upregulated by erythromycin (26). Our data demonstrated that in M. abscessus, *eis2* was within the WhiB7 regulon. *eis2* transcription was induced ∼8-fold by clarithromycin in a WhiB7-dependent manner (Fig. 3), but not by amikacin. Independent studies of M. abscessus cultures growing in a different medium carried out in Ghosh lab at the Wadsworth Center have shown that amikacin can induce *whiB7*, but the response was much weaker than that of other antibiotics tested; higher concentrations were required, the fold induction was lower, and the response was delayed (41). The M. tuberculosis genome does not contain *eis2*; therefore, WhiB7 is linked to amikacin resistance in M. abscessus but not in M. tuberculosis.

Divergence of resistance in these two species reflects evolutionary selective pressures in different niches, as human pathogens, or in environmental communities. Eis belongs to a family of ubiquitous acetyltransferases (GNATs) that have remarkably flexible substrate specificities (42). The Eis protein in M. tuberculosis (Rv2416c) acetylates antibiotics having different structures (aminoglycosides and capreomycin), histone-like proteins that fold its chromosome (43), as well as at least one host protein (44). This allows it not only to provide aminoglycoside and capreomycin resistance, but it also allows the enhancement of intracellular survival (*eis*) in macrophages. However, disruption of *eis* in M. tuberculosis (21) does not alter amikacin resistance. Disruption of *eis*2 increases sensitivity of M. abscessus to capreomycin as well as a range of other aminoglycosides (32). Phylogenetic analysis of *eis*2 indicates that it does not cluster with the mycobacterial *eis* genes. Interestingly, it clusters with homologs found in Streptomyces species (32, 45), bacteria classified together with mycobacteria as members of the Actinobacteria taxon. Streptomyces spp. inhabit soil environments throughout the world and are best known as nonpathogenic producers of the majority of known antibiotics, including macrolides, aminoglycosides, and capreomycin. The fact that Streptomyces genomes carry functional *whiB7* homologs (20) suggests that the *eis*2 may have served to provide resistance to antibiotics produced by these organisms and was retained in M. abscessus.

Our studies revealed that WhiB7 activates resistance to three of the five commonly used M. abscessus drugs, clarithromycin, amikacin, and tigecycline, but not imipenem or cefoxitin, and suggest that it has a central role in treatment failure. We demonstrated that clarithromycin preexposure increased resistance not only to itself but also to amikacin and other aminoglycosides. A wide variety of compounds inhibiting translation (including lincosamides, tetracyclines, aminoglycosides, and macrolides) or other functions (including fluoroquinolones and acivicin) are *whiB7* inducers in M. smegmatis (18). In addition to antibiotics, signals for *whiB7* expression in other mycobacteria include palmitic acid, lung surfactant, iron restriction, sputum, and macrophage infection (20, 26, 46–48). Exposure of M. abscessus to any of these conditions during lung infections may induce *whiB7* and the antibiotic resistance functions it upregulates. The importance of WhiB7-controlled *erm*(41) expression can be seen directly by comparing treatment outcomes with M. abscessus subspecies that do not have a functional Erm(41). Erm(41) is responsible for constitutive macrolide and inducible macrolide resistance in M. abscessus (28). However, M. abscessus can be split into subspecies which do (M. abscessus subsp. abscessus T28 and M. abscessus subsp. bolletii) or do not (M. abscessus subsp. abscessus C28 and M. abscessus subsp. massiliense) contain a functional *erm*(41) (49). Treatment success of M. abscessus subsp. massiliense is 70%, compared to 41% to 35% in M. abscessus with a functional *erm*(41) gene, emphasizing the importance of WhiB7-induced *erm*(41) expression on negative M. abscessus treatment outcome.

Our *in vitro* data suggest that clarithromycin and amikacin, which are front-line coadministered drugs, may have antagonistic effects during treatment. An effective concentration of i.v.-administered amikacin in the lungs may not be achievable in patients whose therapy includes clarithromycin, compounding the issue with Erm(41)-mediated macrolide resistance. This implies that WhiB7-mediated inducible antibiotic resistance decreases the clinical effectiveness of 2 of the 3 antibiotics used in M. abscessus triple therapy. Furthermore, mutations in the M. tuberculosis *whiB7* locus can cause constitutive expression of *whiB7* and lead to increased expression of its resistance regulon (50). Similar mutations in M. abscessus may provide clinically relevant antibiotic-resistant mutants. Together, our data argue against coadministering clarithromycin and i.v. amikacin, since these drugs can be antagonistic. However, if alternative antibiotics cannot be identified, amikacin should only be given by inhalation, yielding a much higher *C*_max_ (∼970 mg/liter) (51) and thus overcoming induced amikacin resistance. Our studies suggest that inhibitors of WhiB7 might increase killing by macrolides, tigecycline, and aminoglycosides, thereby minimizing the spread of high-level resistance.

## MATERIALS AND METHODS

### Bacterial strains.

All cloning was done in Escherichia coli DH5α grown in LB broth supplemented with 50 mg/liter kanamycin, 100 mg/liter ampicillin, or 50 μg/ml apramycin where appropriate. All M. abscessus strains used in these studies (described in Table 4) were classified as Mycobacterium abscessus subsp. abscessus based on 16S and *hps65* gene sequencing. ATCC 19977 was purchased from the ATCC, and clinical M. abscessus strains were obtained from Patrick Tang at the British Columbia Centre for Disease Control. All M. abscessus strains were grown in Mueller-Hinton II (MHII) broth supplemented with 0.05% tyloxapol at 37°C in rolling test tubes, or in flasks shaking at 200 rpm. MHII was supplemented with 50 mg/liter kanamycin, 50 mg/liter apramycin, or 100 mg/liter zeocin when appropriate.

**TABLE 4:** Strain descriptions

Strain	Description^*a*^	Reference or source
M. abscessus	Mycobacterium abscessus (ATCC 19977) strain containing pJV53-zeo; Zeo^r^	53
M. abscessus strain 1	Rough clinical strain isolated from sputum	This study
M. abscessus strain 2	Rough clinical strain isolated from sputum	This study
M. abscessus strain 3	Rough clinical strain isolated from hand ulcer	This study
M. abscessus strain 4	Rough clinical strain isolated from sputum	This study
M. abscessus strain 5	Rough clinical strain isolated from bone joint abscess	This study
M. abscessus strain 6	Rough clinical strain isolated from sputum	This study
Δ*whiB7* mutant	M. abscessus with deletion of *whiB7* (MAB_3508c) from bp 80–131; Kan^r^^,^^*b*^	This study
Δ*aac(2′)* mutant	M. abscessus with deletion of *aac(2′)* (MAB_4395) from bp 200–300; Kan^r^^,^^*b*^	This study
Δ*tap* mutant	M. abscessus with deletion of *tap* (MAB_1409c) from bp 551–560; Kan^r^^,^^*b*^	This study
Δ*whiB7*-C mutant	Δ*whiB7* (Kan^r^^*b*^) with pMV261 expressing MAB_3509c (upstream gene in operon) and *whiB7* gene from native promoter; Apr^r^^*c*^	This study

a Zeo^r^, zeomycin resistance; Kan^r^, kanamycin resistance; Apr^r^, apramycin resistance.

b Resistance cassette from pMV261 (52).

c Resistance cassette from pT10full.

### Cloning.

PCRs were performed using Q5 high-fidelity DNA polymerase (catalog no. M0491S; New England BioLabs), according to the manufacturer's instructions. All restriction enzymes were purchased from New England BioLabs and used according to the manufacturer's instructions. Ligations were done with T4 DNA ligase (catalog no. 15224-041; Invitrogen) overnight at 16°C. The primers used are listed in Table S1. Blunt-end reactions were done with the Klenow fragment (catalog no. M0210S; New England BioLabs), as per the manufacturer's instructions.

### Construction of *whiB7*, *tap*, and *aac*(*2′*) mutants.

*whiB7*, *tap*, and *aac*(*2′*) genes were PCR amplified with primers whiB7-FW and whiB7-RV, tap-FW and tap-RV, and *aac*(*2′*)-FW and *aac*(*2′*)-RV, respectively. The resulting products were A-tailed with *Taq* DNA polymerase, and the fragments were ligated into pGEM-T Easy to create pGem-whiB7, pGem-tap, and pGem-*aac*(*2′*). Plasmids were PCR amplified using primers whiB7-iPCR-FW and whiB7-iPCR-RV, tap-iPCR-FW and tap-iPCR-RV, and *aac*(*2′*)-iPCR-FW and *aac*(*2′*)-iPCR-RV that added terminal unique HindIII (forward [FW] primers) and StuI (reverse [RV] primers) sites. The PCR products were then digested with HindIII and StuI. An *aph* kanamycin resistance gene, isolated from pMV261 (52) using StuI and HindIII, was then ligated into StuI/HindIII-digested pGem-whiB7, pGem-tap, and pGem-*aac*(*2′*) PCR products.

Fragments containing *whiB7*, *tap*, or *aac*(*2′*) genes disrupted with the kanamycin resistance gene were isolated from the pGEM backbone with SphI and SacI to yield linear DNA. Linear DNA products were electroporated into M. abscessus containing the pJV53-zeo plasmid (53), and the resulting transformants were selected for on MHII-kanamycin agar plates. Gene disruptions were confirmed using primers whiB7-OS-FW and whiB7-OS-RV, tap-OS-FW and tap-OS-RV, and *aac*(*2′*)-OS-FW and *aac*(*2′*)-OS-RV.

### Construction of pWhiB7-C complementing plasmid.

To complement the *whiB7* mutant, *whiB7*, including ∼500 bp of its upstream region (to encompass the native promoter), was PCR amplified from M. abscessus genomic DNA using primers WhiB7-C-FW and WhiB7-C-RV. The resulting PCR product was digested with HindIII and PstI and cloned into a similarly digested modified pMV261 backbone (using the plasmid pFB7 [19]) to construct pMV261-whiB7.

To remove the pMV261 kanamycin resistance gene and introduce the apramycin resistance gene, the plasmid pMV261-whiB7 was digested with SpeI and blunted, followed by HindIII digestion. From pT10full (J. Burian, unpublished data), the apramycin resistance gene was removed by digestion with NcoI, whose end was blunted, followed by HindIII digestion. The resulting HindIII/blunt digestion products were ligated to construct pWhiB7-C (plasmid map and sequence are shown in Fig. S1).

### MIC determination.

M. abscessus was inoculated into MHII medium and grown for 48 h in rolling test tubes at 37°C to a final optical density at 600 nm (OD_600_) of 2 to 5. For preinduction, cultures were diluted to an OD_600_ of 0.01 in 3 ml of MHII medium containing 0.05 to 0.1 mg/liter clarithromycin, 1.6 mg/liter or 0.4 mg/liter amikacin, 0.4 mg/liter or 0.1 mg/liter tigecycline, or 50 mg/liter or 12.5 mg/liter chloramphenicol for 24 h at 37°C in rolling test tubes. After 24 h, cultures were diluted to an OD_600_ of 0.005, and 100 μl was added to 100 μl of MHII medium containing serial 2-fold dilutions of antibiotics in 96-well plates (product no. 3370; Costar). Plates were then incubated for 48 h or 7 days, followed by the addition of 30 μl of 10 mg/100 ml resazurin/water. Plates were incubated for an additional 24 h, and wells that remained blue were deemed negative for growth. Wells that turned pink were assigned as growth.

### CFU analysis.

M. abscessus was inoculated into MHII medium and grown in rolling test tubes at 37°C for 48 h to a final OD_600_ of 2 to 5. Cultures were then diluted to an OD_600_ of 0.005 in 3 ml of MHII medium in test tubes. For preinduction experiments, M. abscessus was incubated for 2.5 h with 0.1 mg/liter or 20 mg/liter clarithromycin before supplementing cultures with a range of amikacin concentrations (3 to 32 mg/liter). For CFU kill curves, a log-phase (to OD_600_ 0.7 to 1.5) culture was diluted to an OD_600_ of 0.005, and 3-ml volumes were added to test tubes with appropriate concentrations of amikacin, tobramycin, or clarithromycin. At specified times, 100 μl of culture was removed from each tube, and serial 10-fold dilutions were done. Ten microliters of each dilution was spotted onto MHII agar plates, which were then incubated at 37°C. Colonies were counted after 5 days.

### RNA extraction.

M. abscessus was inoculated into MHII medium and grown in shaking flasks at 37°C for 48 h to an OD_600_ of 0.5 to 0.6. Cultures were split, and ½ the MIC clarithromycin or amikacin was added (WT, 0.1 mg/liter clarithromycin or 1.6 mg/liter amikacin; Δ*whiB7* mutant, 0.05 mg/liter clarithromycin) for comparison with an untreated control. The cultures were then incubated for 3 h shaking at 37°C. RNA was extracted as previously described (18).

### Quantitative PCR.

Synthesis of cDNA and quantitative PCR (qPCR) analysis were previously described (18). Generation of cDNA was done with qScript cDNA synthesis kit (catalog no. 95047-100; Quanta), as per the manufacturer's instructions, with a total of 100 ng of isolated RNA. The Bioline SensiFAST SYBR No-ROX kit (BIO-98005) was used for qPCR analysis using a Bio-Rad Opticon 2. The primers used for *whiB7* were whiB7-qPCR-FW and whiB7-qPCR-RV; for *erm*(41), the primers were erm-qPCR-FW and erm-qPCR-RV; for *aac*(*2′*), the primers were aac(2′)-qPCR-FW and aac(2′)-qPCR-RV; and for *eis2*, the primers were eis2-qPCR-FW and eis2-qPCR-RV. Concentrations were calculated against a standard curve of genomic DNA dilutions paired with the same primers. Values were standardized to an internal control, *sigA*, which was measured using the primers sigA-qPCR-FW and sigA-qPCR-RV. Fold increase was calculated by comparison to a nontreated control run in parallel.

## Supplementary Material

Supplemental material
