# Supplementary material for: Antagonism between Front-Line Antibiotics Clarithromycin and Amikacin in the Treatment of Mycobacterium abscessus Infections Is Mediated by the whiB7 Gene
Source: Antimicrob Agents Chemother. 2017 Oct 24;61(11):e01353-17. doi: 10.1128/AAC.01353-17 (PMC5655113; doi:10.1128/AAC.01353-17)
Supplement: Supplemental material [file supp_61_11_e01353-17__index.html]

Supplemental material 

# Antagonism between Front-Line Antibiotics Clarithromycin and Amikacin in the Treatment of Mycobacterium abscessus Infections Is Mediated by the *whiB7* Gene

## Supplemental material

- Supplemental file 1 -

  Supplemental Figures S1 to S3 and Tables S1 to S3

  PDF, 826K
