# Supplementary material for: Antagonism between Front-Line Antibiotics Clarithromycin and Amikacin in the Treatment of Mycobacterium abscessus Infections Is Mediated by the whiB7 Gene
Source: Antimicrob Agents Chemother. 2017 Oct 24;61(11):e01353-17. doi: 10.1128/AAC.01353-17 (PMC5655113; doi:10.1128/AAC.01353-17)
Supplement: Supplemental material [file AAC.01353-17_zac011176656s1.pdf]

Supplemental Figure S1.

Map of pWhiB7-C

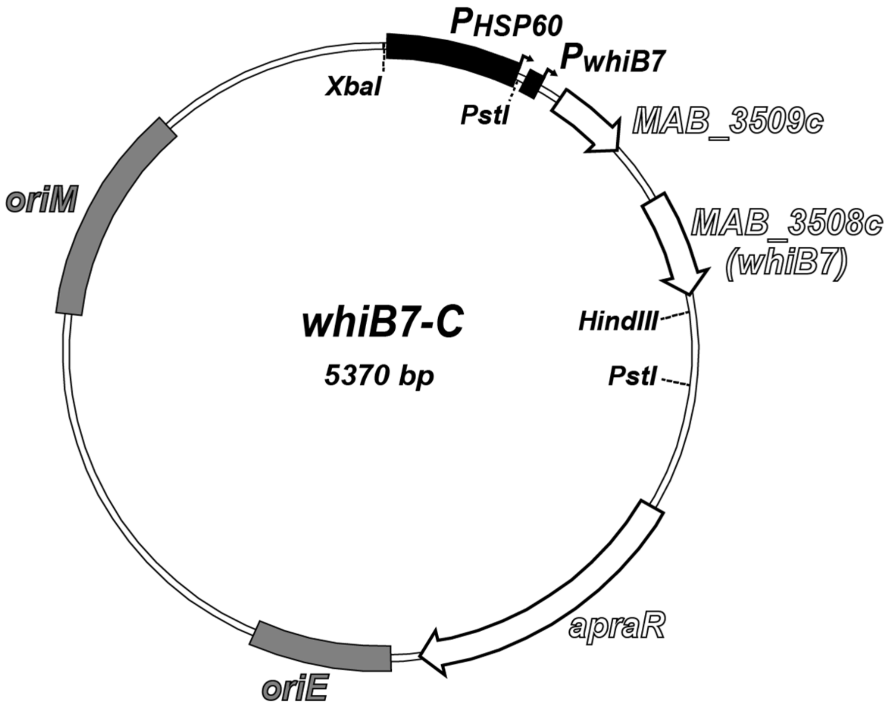

Supplemental Figure S1. *whiB7* (MAB_3508c) sequence.

```
ID    whiB7-C   standard; circular DNA;    ; 5370 BP.
FH    Key             Location/Qualifiers
 FH
 FT   CDS             508..720
 FT                   /vntifkey="4"
 FT                   /label=MAB_3509c
 FT   CDS             890..1174
 FT                   /vntifkey="4"
 FT                   /label=MAB_3508c\(whiB7)
 FT   rep_origin      4134..4734
 FT                   /vntifkey="33"
 FT                   /label=oriM
 FT   promoter        7..377
 FT                   /vntifkey="30"
 FT                   /label=PHSP60
 FT                   /note="HSP60 promoter region"
 FT   CDS             1790..2574
 FT                   /vntifkey="4"
 FT                   /label=apraR
 FT   rep_origin      2654..3034
 FT                   /vntifkey="33"
 FT                   /label=oriE
 FT   promoter        399..445
 FT                   /vntifkey="30"
 FT                   /label=PwhiB7
 SQ   Sequence 5370 BP; 1107 A; 1560 C; 1642 G; 1061 t;

      ctagacggtg accacaacgc gcccgctttg atcggggacg tctgcggccg accatttacg        60
      ggtcttgttg tcgttggcgg tcatgggccg aacatactca cccggatcgg agggccgagg       120
      acaaggtcga acgaggggca tgacccggtg cggggcttct tgcactcggc ataggcgagt       180
      gctaagaata acgttggcac tcgcgaccgg tgagtgctag gtcgggacgg tgaggccagg       240
      cccgtcgtcg cagcgagtgg cagcgaggac aacttgagcc gtccgtcgcg ggcactgcgc       300
      ccggccagcg taagtagcgg ggttgccgtc acccggtgac cccgtttca tccccgatcc       360
      ggaggaatca cttcgcactg caggtatcgc cgcaggtcaa aaataacttg tcaaccgata       420
      ggtgaccttt tactctgggc ttcaggaatt cgcgaggatt ccaaaccaag acctgttcac       480
      gtgcgagtag acggaaggag gagcgaaatg aacacaccca ccaattacgg agacaccttg       540
      tggcgtgatg cccatggtgc ctttgtcgtg ttgccgatcg ctccgccgtc gctccagacg       600
      cgtgccgccg ccaagccggc ggtagctgcc gtgttgatgg ccggctcggc ttccgcgatt       660
      aagcaccgcg ccgccgtcgc agccgcgaat gtcgcgtccg tgaatagggg atacatctag       720
      gtatccccac aaccgcctcc caggccacgg acccgacacc accgggatcc gtggccacat       780
      ttgtttgagc cccaaacctc aagcgtggtt ccggatctcg atcgataaga caccgatacg       840
      agaccagcag ttttacgagg aaccgaagag gaagcaggtg aacggacaca tgatgaccgt       900
      tgaagtggag gcccgaagac tcgcgctgcc gtgccacgtc gcggacgcgg acctgtggtt       960
      cgcggaaagc cccgccgacc tggagcgggc gaaggcgctg tgcgcggact gcccgatccg      1020
      gtcgcagtgc ctggccgctg cgctggaccg cgcagagccg tggggagtgt ggggcggtga      1080
      gattcttgag caggggacca ttgtggcgcg caagcggccg cgtggacgcc cgcgcaagaa      1140
      cccactgccc gacgccgaca ccgccgcggc atgacgccgc cggccaagaa ggttgtaggg      1200
      cctgaagctt ttaaggttta acggttgtgg acaacaagcc agggatgtaa cgcactgaga      1260
      agcccttaga gcctctcaaa gcaattttga gtgacacagg aacacttaac ggctgacatg      1320
      ggaattcttg aagacgaaag ggcctcgtga tacgcctatt tttataggtt aatgtcatga      1380
      taataatggt ttcttagacg tcaggtggca cttttcgggg ttaaagggct gcaggaattc      1440
      gatagcttgc tgacgccgtt ggatacacca aggaaagtct acacgaaccc tttggcaaaa      1500
      tcctgtatat cgtgcgaaaa aggatggata taccgaaaaa atcgctataa tgaccccgaa      1560
      gcagggttat gcagcggaaa atgcagctca cggtaactga tgccgtattt gcagtaccag      1620
      cgtacggccc acagaatgat gtcacgctga aaatgccggc ctttgaatgg gttcatgtgc      1680
      agctccataa gcaaaagggg atgataagtt tatcaccacc gactatttgc aacagtgccg      1740
      ttgatcgtgc tatgatcgac tgatgtcatc agcggtggag tgcaatgtcg tgcaatacga      1800
      atggcgaaaa gccgagctca tcggtcagct tctcaacctt ggggttaccc ccggcggtgt      1860
      gctgctggtc cacagctcct tccgtagcgt ccggcccctc gaagatgggc cacttggact      1920
      gatcgaggcc ctgcgtgctg cgctgggtcc gggagggacg ctcgtcatgc cctcgtggtc      1980
      aggtctggac gacgagccgt tcgatcctgc cacgtcgccc gttacaccgg accttggagt      2040
```

```
tgtctctgac acattctggc gcctgccaaa tgtaaagcgc agcgcccatc catttgcctt 2100
cgcggcagcg gggccacagg cagagcagat catctctgat ccattgcccc tgccacctca 2160
ctcgcctgca agcccggtcg cccgtgtcca tgaactcgat gggcaggtac ttctcctcgg 2220
cgtgggacac gatgccaaca cgacgctgca tcttgccgag ttgatggcaa aggttcccta 2280
tggggtgccg agacactgca ccattcttca tgatggcaag ttggtacgcg tcgattatct 2340
cgagaatgac cactgctgtg agcgctttgc cttggcggac aggtggctca aggagaagag 2400
ccttcagaag gaaggtccag tcggtcatgc ctttgctcgg ttgatccgct cccgcgacat 2460
tgtggcgaca gccctgggtc aactgggccg agatccgttg atcttcctgc atccgccaga 2520
ggcgggatgc gaagaatgcg atgccgctcg ccagtcgatt ggctgagctc ataagttcct 2580
attccgaagt tcctattctc tagtcgaggg ggggcccgag cttagctggc tagcgagtca 2640
tgaggtcacc atgctagttc cactgagcgt cagaccccgt agaaaagatc aaaggatctt 2700
cttgagatcc tttttttctg cgcgtaatct gctgcttgca aacaaaaaaa ccaccgctac 2760
cagcggtggt ttgtttgccg gatcaagagc taccaactct ttttccgaag gtaactggct 2820
tcagcagagc gcagatacca aatactgtcc ttctagtgta gccgtagtta ggccaccact 2880
tcaagaactc tgtagcaccg cctacatacc tcgctctgct aatcctgtta ccagtggctg 2940
ctgccagtgg cgataagtcg tgtcttaccg ggttggactc aagacgatag ttaccggata 3000
aggcgcagcg gtcgggctga acggggggtt cgtgcacaca gcccagcttg gagcgaacga 3060
cctacaccga actgagatac ctacagcgtg agcattgaga aagcgccacg cttcccgaag 3120
ggagaaaggc ggacaggtat ccggtaagcg gcagggtcgg aacaggagag cgcacgaggg 3180
agcttccagg gggaaacgcc tggtatcttt atagtcctgt cgggtttcgc cacctctgac 3240
ttgagcgtcg atttttgtga tgctcgtcag gggggcggag cctatggaaa aacgccagca 3300
acgcggcctt tttacggttc ctggcctttt gctggccttt tgctcacatg ttctttcctg 3360
cgttatcccc tgattctgtg gataaccgta ttaccgcctt tgagtgagct gataccgctc 3420
gccgcagccg aacgaccgag cgcaacgcgt gagcccacca gctccgtaag ttcgggtgct 3480
gtgtggctcg tacccgcgca ttcaggcggc agggggtcta acgggtctaa ggcggcgtgt 3540
acggccgcca cagcggctct tagcggcccg gaaacgtcct cgaaacgacg catgtgttcc 3600
tcctggttgg tacaggtggt tgggggtgct cggctgtcgc tggtgtttca tcatcagggc 3660
tcgacgggag agcgggggag tgtgcagttg tggggtggcc cctcagcgaa atatctgact 3720
tggagctcgt gtcggaccat acaccggtga ttaatcgtgg tttattatca agcgtgagcc 3780
acgtcgccga cgaatttgag cagctctggc tgccgtactg gtccctggca agcgacgatc 3840
tgctcgaggg gatctaccgc caaagccgcg cgtcggccct aggccgccgg tacatcgagg 3900
cgaacccaac agcgctggca aacctgctgg tcgtggacgt agaccatcca gacgcagcgc 3960
tccgagcgct cagcgcccgg gggtcccatc cgctgcccaa cgcgatcgtg ggcaatcgcg 4020
ccaacggcca cgcacacgca gtgtgggcac tcaacgcccc tgttccacgc accgaatacg 4080
cgcggcgtaa gccgctcgca tacatggcgg cgtgcgccga aggccttcgg cgcgccgtcg 4140
atggcgaccg cagttactca ggcctcatga ccaaaaaccc cggccacatc gcctgggaaa 4200
cggaatggct ccactcagat ctctacacac tcagccacat cgaggccgag ctcggcgcga 4260
acatgccacc gccgcgctgg cgtcagcaga ccacgtacaa agcggctccg acgccgctag 4320
ggcggaattg cgcactgttc gattccgtca ggttgtgggc ctatcttccc gccctcatgc 4380
ggatctacct gccgacccgg aacgtggacg gactcggccg cgcgatctat gccgagtgcc 4440
acgcgcgaaa cgccgaattt ccgtgcaacg acgtgtgtcc cggaccgcta ccggacagcg 4500
aggtccgcgc catcgccaac agcatttggc gttggatcac aaccaagtcg cgcatttggg 4560
cggacgggat cgtggtctac gaggccacac tcagtgcgcg ccatgcggcc atctcgcgga 4620
agggcgcagc agcgcgcacg gcggcgagca cagttgcgcg gcgcgcaaag tccgcgtcag 4680
ccatggaggc attgctatga gcgacggcta cagcgacggc tacagcgacg gctacaactg 4740
gcagccgact gtccgcaaaa agcggcgcgt gaccgccgcc gaaggcgctc gaatcaccgg 4800
actatccgaa cgccacgtcg tccggctcgt ggcgcaggaa cgcagcgagt ggttcgccga 4860
gcaggctgca cgccgcgaac gcatccgcgc ctatcacgac gacgagggcc actcttggcc 4920
gcaaacggcc aaacatttcg ggctgcatct ggacaccgtt aagcgactcg gctatcgggc 4980
gaggaaagag cgtgcggcag aacaggaagc ggctcaaaag gcccacaacg aagccgacaa 5040
tccaccgctg ttctaacgca attggggagc gggtgtcgcg ggggttccgt gggggttcc 5100
gttgcaacgg gtcggacagg taaaagtcct ggtagacgct agttttctgg tttgggccat 5160
gcctgtctcg ttgcgtgttt cgttgcgtcc gttttgaata ccagccagac gagacggggt 5220
tctacgaatc ttggtcgata ccaagccatt tccgctgaat atcgtggagc tcaccgccag 5280
aatcggtggt tgtggtgatg tacgtggcga actccgttgt agtgcttgtg gtggcatccg 5340
tggcgcggcc gcggtaccag atctttaaat 5370
```

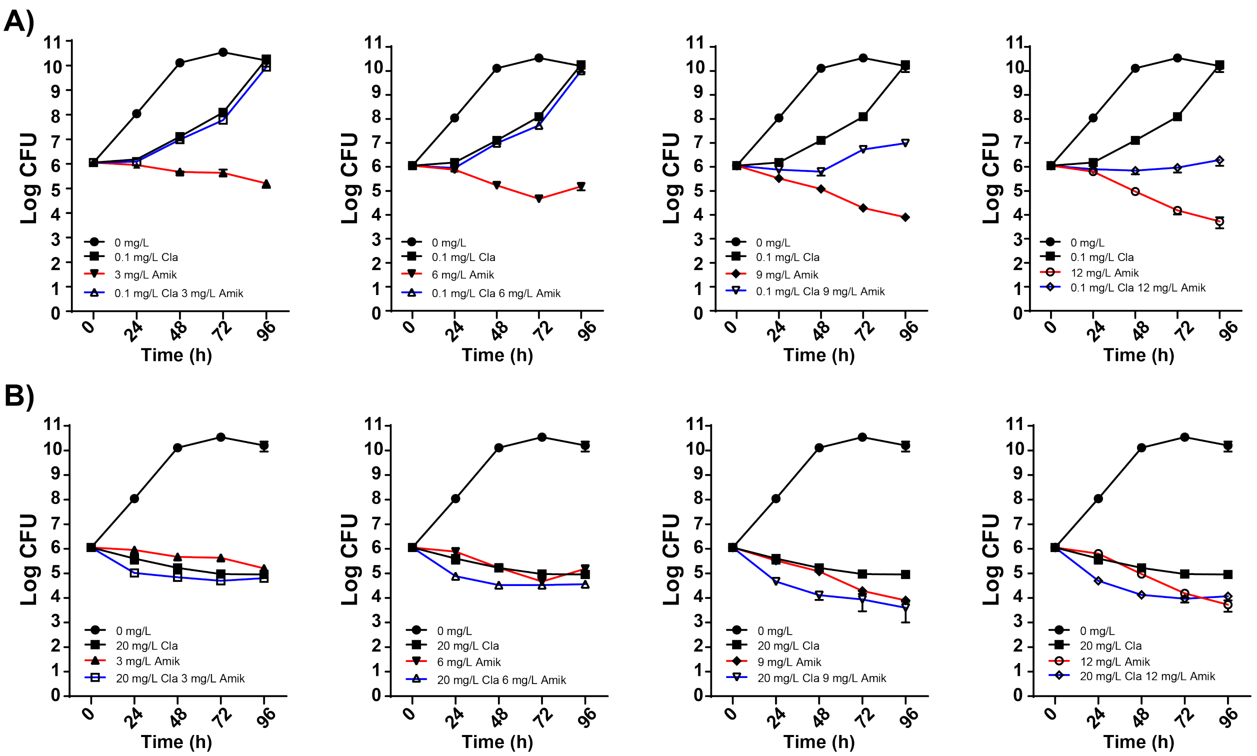

Supplemental Figure S2. Simultaneous clarithromycin and amikacin treatment of *MAB*. *MAB* was incubated with clarithromycin at **A)** ½ MIC (0.1 mg/L) or **B)** 100X MIC (20 mg/L) followed by immediate addition of a range of amikacin concentrations (3-12 mg/L). CFUs were monitored at 24 h intervals. Data points are the means of three replicates with error bars indicating standard deviation.

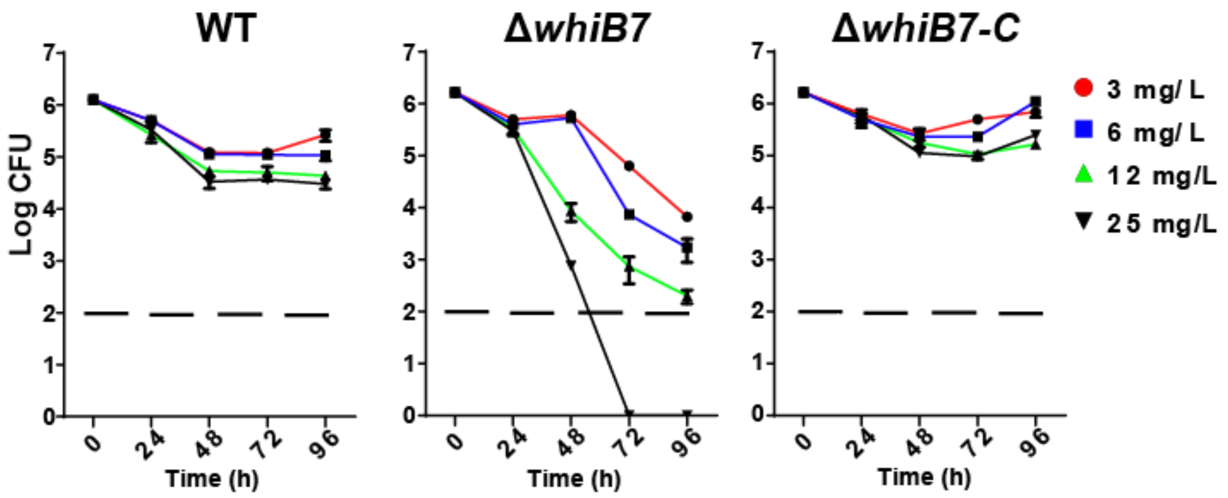

Supplemental Figure S3: Effect of clinically-relevant clarithromycin concentrations on *MAB* wild type, Δ *whiB7* and Δ *whiB7*-C derivatives. Strains were incubated with clarithromycin concentrations found in the lung. CFUs were monitored at 24 h intervals. Dashed lines represent the limit of detection. Data points are the mean of 3 replicates with standard deviation given as error bars. The data represents 3 independent experiments.

Supplemental Table S1. Primers used in this study

Primer	Sequence (5’ to 3’)
whiB7-FW	GAAATGAACACACCCACCAATTACG
whiB7-RV	GATCAGGTGATGGATTACATCAGAC
aac(2)-FW	GATCCACGGAAACTGGATAGTC
aac(2)-RV	GTTGTCTGACGAGGCGAC
tap-FW	GATTCGCCGACTATCAATCAGGTGAAT
tap-RV	GTTGGTTCCTTGTCGGGCTG
WhiB7-iPCR-RV	GCATAGGCCTCTTTCCGCGAACCACAGGTC
WhiB7-iPCR-FW	GCATAAGCTTGTCGCAGTGCCTGGC
aac(2)-iPCR-RV	GCATAGGCCTATGAGTATGCCGTGGTACCAG
aac(2)-iPCR-FW	GCATAAGCTTCGTGGAAACGGACTTGGTAC
tap-iPCR-RV	CGATAAGCTTCTTCGTCGTGTCGATCATCG
tap-iPCR-FW	GCATACTAGTGTGACCCACATGGTGTTGAC
whiB7-OS-FW	GGTAGCTGCCGTGTTGAT
whiB7-OS-RV	GTCGACCGACCTGTTGGAC
aac(2)-OS-FW	CCGATCCCGATGCCTTG
aac(2)-OS-RV	CGTGTTCTACATCGGTAGTTGTCTG
tap-OS-FW	GATCCCGATCTGGAGGC
tap-OS-RV	CCCGTGATCAGTGCAG
whiB7-C-FW	CGATCTGCAGGTATCGCCGCAGGTCAAAAATAAC
whiB7-C-RV	GCATAAGCTTCAGGCCCTACAACCTTCTTG
whiB7-qPCR-FW	ACCTCAAGCGTGGTTCCG
whiB7-qPCR-RV	CCTCCACTTCAACGGTCATC
aac(2)-qPCR-FW	CTGTGTCCAATATGCCAACC
aac(2)-qPCR-RV	GAATGCCTCAATGAGTAATTCAC
erm-qPCR-FW	GGAGTTCGTTGTGGATCTGG
erm-qPCR-RV	AAACCGTGAACGAAGGTGTC
eis2-qPCR-FW	GAGGTCAACCGCAAATTCAC
eis2-qPCR-RV	CACGACATGACGGCTGAAC
sigA-qPCR-FW	CACAAAGGGTTACAAGTTCTCG
sigA-qPCR-RV	GCTTGTTGATGACCTCGACC

Supplemental Table S2. The effect of *whiB7* deletion on the resistance profile of *MAB*.

Drug	MIC (mg/L)	
	WT	Δ*whiB7*
Imipenem	6.3	6.3
Cefoxitin	50	50
Doxycycline	>100	>100
Tetracycline	>100	>100
Clindamycin	12.5	25
Levofloxacin	3.1	3.1
Moxifloxacin	1.6	1.6
Rifabutin	6.3	6.3
Rifamycin	>100	>100
Rifamycin SV	25	25
Isoniazid	>100	>100
Ethambutol	>100	>100

Supplemental Table S3: Roles of *tap* and *eis* in aminoglycoside resistance.

Drug	Amikacin MIC (mg/L)					
	0 mg/L Clarithromycin			½ MIC Clarithromycin		
	WT	Δ *aac(2')*	Δ*tap*	WT	Δ *aac(2')*	Δ*tap*
Amikacin	3.1	3.1	3.1	12.5	12.5	12.5
Tobramycin	6.3	0.4	6.3	25	0.4	25
Gentamicin	6.3	3.1	3.1	25	6.3	12.5
Sisomicin	3.1	0.8	3.1	12.5	0.8	12.5

Values are the medians of 3 experiments.
